# Clinical and epidemiological characteristics of patients hospitalized for toxocariasis in Japan: A nationwide analysis using the diagnosis procedure combination database

**DOI:** 10.1016/j.parepi.2026.e00546

**Published:** 2026-09-17

**Authors:** Takafumi Matsunaga, Naoaki Ohkubo, Kei Yamasaki, Masahiro Tahara, Kei Nakamura, Hiroaki Ikegami, Konomi Sennari, Takanobu Jotatsu, Chinatsu Nishida, Makoto Okawara, Yoshihisa Fujino, Shinya Matsuda, Kiyohide Fushimi, Hiroshi Mukae, Kazuhiro Yatera

**Affiliations:** a Department of Respiratory Medicine, University of Occupational and Environmental Health, Japan, Kitakyushu, Japan; b Department of Environmental Epidemiology, Institute of Industrial Ecological Sciences, University of Occupational and Environmental Health, Japan, Kitakyushu, Japan; c Department of Respiratory Medicine, Wakamatsu Hospital of the University of Occupational and Environmental Health, Japan, Kitakyushu, Japan; d Department of Environmental Health Engineering, Institute of Industrial Ecological Sciences, University of Occupational and Environmental Health, Japan, Kitakyushu, Japan; e Department of Preventive Medicine and Community Health, University of Occupational and Environmental Health, Japan, Kitakyushu, Japan; f Department of Nursing, Fukuoka International University of Health and Welfare, Fukuoka, Japan; g Department of Health Policy and Informatics, Institute of Science Tokyo, Tokyo, Japan; h Department of Respiratory Medicine, Nagasaki University Graduate School of Biomedical Sciences, Nagasaki, Japan

**Keywords:** Administrative database, Diagnosis procedure combination, Nationwide study, Ocular toxocariasis, Toxocariasis, Visceral larva migrans

## Abstract

Toxocariasis is a zoonotic helminth infection caused by *Toxocara canis* or *Toxocara cati*. While it has traditionally been regarded as a pediatric soil-transmitted disease, recent reports from Japan suggest that an increasing number of adult-onset cases are potentially associated with foodborne transmission. Ocular involvement in adults can also result in severe visual impairment. However, nationwide data describing the epidemiological and clinical characteristics of hospitalized patients in Japan are limited. We conducted a nationwide retrospective study using the Japanese Diagnosis Procedure Combination database from the fiscal years 2014 through 2020. Hospitalized patients with toxocariasis were identified using ICD-10 code B83.0. Demographic characteristics, organ involvement, geographic distribution, and treatment patterns were descriptively analyzed. Forty-one patients with toxocariasis were identified. The median age was 53 years (range, 10–89 years), and 63% of the patients were male. None of the patients were younger than 10 years. Ocular involvement was the most frequent presentation (41%), and ophthalmologic surgery, including pars plana vitrectomy, was performed in 24% of the cases. Patients who were hospitalized were predominantly from the Kinki (32%) and Kyushu–Okinawa (29%) regions. Albendazole was administered to 54% of the patients, with a median inpatient treatment duration of 3 days (inpatient administration only). In Japan, toxocariasis requiring hospitalization predominantly affects middle-aged and older adults and is frequently associated with ocular involvement requiring surgical intervention. The adult-dominant patterns and geographic clustering in Western Japan may reflect region-specific exposure characteristics. Although limited to hospitalized patients and administrative data without diagnostic validation, these findings provide the first nationwide overview of severe toxocariasis requiring hospitalization in Japan and contribute to understanding its clinical and epidemiological profile.

## Graphical abstract

**Figure:**
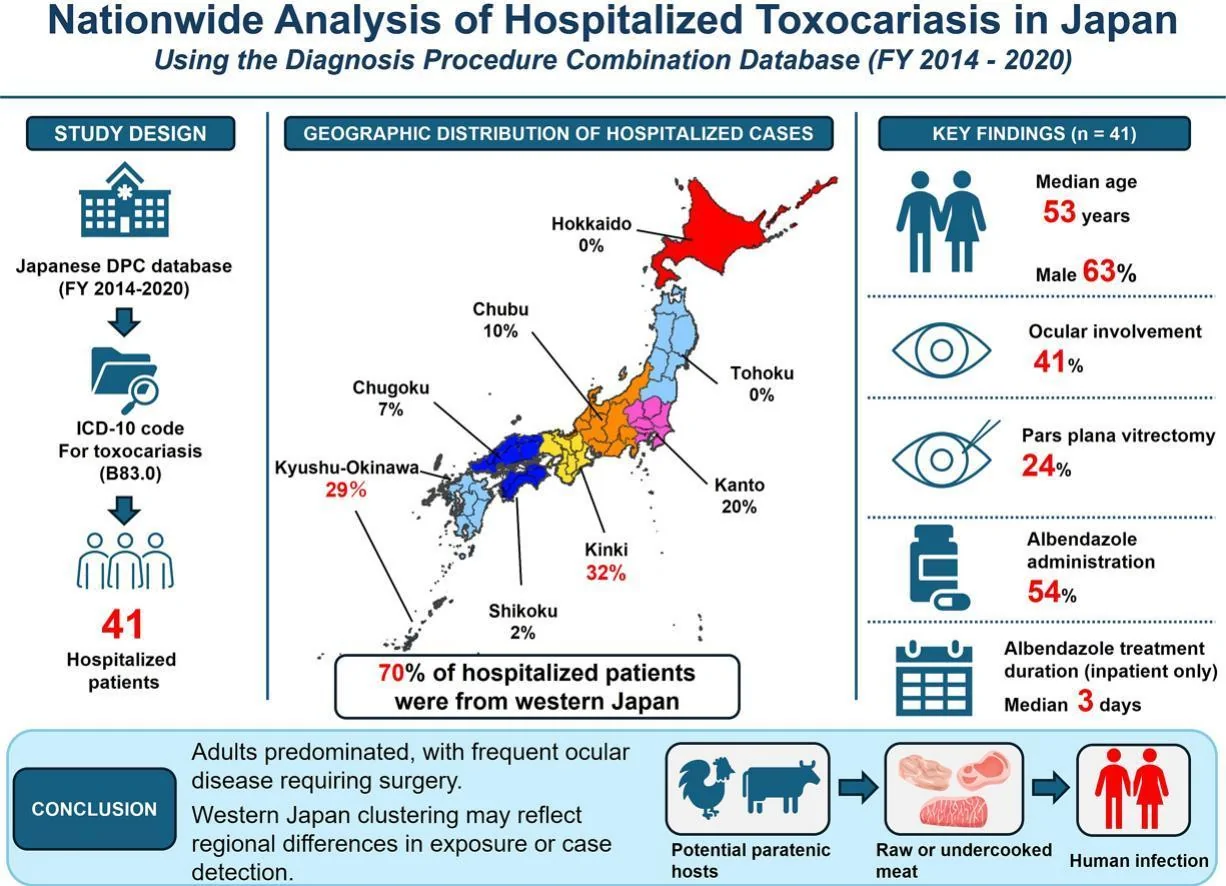

## 1 Introduction

Toxocariasis is a globally distributed zoonotic helminth infection caused by the larvae of *Toxocara canis* or *Toxocara cati*, in which humans serve as accidental hosts. It occurs through the ingestion of embryonated eggs or larvae from contaminated sources. After hatching in the intestine, larvae migrate through various organs, resulting in a spectrum of clinical manifestations collectively known as visceral larva migrans (VLM) and ocular larva migrans (OLM) (Despommier, 2003; Macpherson, 2013). Despite its recognized public health significance, toxocariasis is categorized as a neglected tropical disease and its global burden is likely underestimated (Ma et al., 2018).

Traditionally, toxocariasis has been regarded as a pediatric disease acquired through the accidental ingestion of embryonated eggs present in contaminated soil, particularly in playgrounds and sandboxes (Magnaval et al., 2001). However, emerging evidence suggests that epidemiological patterns in Japan may differ from this classical paradigm. A shift in the transmission route from soil-mediated to foodborne via the ingestion of raw or undercooked paratenic hosts has been described (Akao and Ohta, 2007; Healy et al., 2022). In Japan, toxocariasis has been reported following the consumption of raw chicken liver, and *Toxocara* larvae have been detected in the muscles of farm chickens, supporting the potential role of chickens as paratenic hosts (Morimatsu et al., 2006; Okada et al., 2021). Similar associations between toxocariasis and the consumption of raw meat or liver have also been reported in other East Asian countries, particularly Korea (Choi et al., 2012; Choi et al., 2008). Correspondingly, the affected population appears to have shifted from children to adults and severe adult cases of ocular toxocariasis requiring invasive interventions have been increasingly reported (Wakabayashi et al., 2021).

Although the growing recognition of adult-onset and foodborne toxocariasis in Japan has been suggested, its nationwide epidemiological characteristics remain unclear. Most previous studies have been limited to serological surveys conducted at specialized institutions (Akao and Ohta, 2007) or sporadic case reports from single centers. Nationwide data describing the geographic distribution of patients who have been hospitalized or real-world treatment patterns of cases requiring inpatient management are lacking. Clarifying these is essential for understanding the clinical and public health impact of toxocariasis in Japan. The Diagnosis Procedure Combination (DPC) database has also been used in nationwide epidemiological studies on other parasitic diseases in Japan, demonstrating its utility for characterizing the clinical and geographic features of patients who have been hospitalized (Ikushima et al., 2024; Nakamura et al., 2026; Sakakibara et al., 2026; Shingu et al., 2026).

Therefore, we conducted a nationwide retrospective analysis using the Japanese DPC inpatient database. The aim of this study was to clarify the demographic characteristics, clinical features, geographic distributions, and real-world treatment patterns of patients hospitalized for toxocariasis in Japan.

## 2 Materials and methods

### 2.1 Patients and data source

This retrospective study was conducted using data derived from the Japanese DPC database, a nationwide administrative inpatient database maintained by the Ministry of Health, Labour and Welfare of Japan. We collected these data through the DPC Research Institute and used them for the analysis. The analysis encompassed data from fiscal years (FYs) 2014 through 2020. As the Japanese fiscal year extends from April to March of the subsequent year, the corresponding study period spanned April 1, 2014, through March 31, 2021.

The DPC system has been widely implemented in Japan and is estimated to cover almost all acute care hospitalizations (Hayashida et al., 2021). Hospital reimbursements are determined using a hybrid payment system that combines DPC-based per-diem payments with fee-for-service components. The DPC database includes detailed information on principal diagnoses; comorbidities present at admission; complications arising during hospitalization; and demographic and clinical variables, including age, sex, residential region, length of hospital stay, discharge status, and procedures performed during hospitalization. Diagnoses are recorded in Japanese and coded according to the International Classification of Diseases, 10th Revision (ICD-10) (Matsuda, 2016).

Patients hospitalized for toxocariasis were identified using ICD-10 code B83.0. To minimize duplicate case inclusion, rehospitalizations under different diagnoses were identified and excluded based on the date of birth and sex of patients, and only the first hospitalization during the study period was included in the analysis. The extracted variables included age, sex, residential region, length of hospital stay, height, weight, and body mass index (BMI), when available. The clinical characteristics were further evaluated, including the organs involved, therapeutic interventions (medical and surgical), and in-hospital comorbidities.

### 2.2 Study variables and definitions

Organ involvement was classified as ocular, pulmonary, hepatic, other, or unknown based on the diagnostic entries and procedure records during hospitalization. Ocular involvement was identified based on relevant diagnostic entries and records of ophthalmological procedures, including pars plana vitrectomy or intraocular lens implantation.

Anthelmintic therapy administered during hospitalization was identified from the medication records. Albendazole and diethylcarbamazine use and the duration of administration were evaluated. Ophthalmologic surgical procedures, including pars plana vitrectomy, intraocular lens implantation, and glaucoma filtration surgery, were identified using procedure codes.

Comorbidities recorded during hospitalization were identified from the diagnostic entries and included diabetes mellitus, hypertension, cognitive impairment/dementia, dyslipidemia, arrhythmia, congestive heart failure, and old cerebral infarction.

Geographic distribution was evaluated based on the patients' registered residential prefectures and categorized according to the standard administrative regions of Japan: Hokkaido, Tohoku, Kanto, Chubu, Kinki, Chugoku, Shikoku, and Kyushu–Okinawa. Temporal trends were assessed by calculating the annual hospitalization numbers stratified by sex. Age distribution was examined using 10-year age categories (<10, 10–19, 20–29, 30–39, 40–49, 50–59, 60–69, 70–79, and ≥ 80 years).

### 2.3 Statistical analysis

The patient characteristics were summarized using descriptive statistics. Continuous variables are presented as mean (standard deviation) or median (minimum–maximum), as appropriate, based on the distribution. Categorical variables were expressed as numbers and percentages. Given the small sample size and the descriptive nature of this nationwide study, no inferential statistical analyses were performed. The observed demographic and geographic patterns were therefore interpreted descriptively without formal hypothesis testing. All statistical analyses were performed using the SPSS software (version 27; IBM Corp., Armonk, NY, USA).

### 2.4 Ethical approval

The study protocol was reviewed and approved by our Institutional Review Board (approval number: R4–045). The requirement for informed consent was waived because of the retrospective nature of the study and the use of anonymized administrative data.

## 3 Results

### 3.1 Patient characteristics

Between FY 2014 and FY 2020, toxocariasis (ICD-10 code B83.0) was documented in 50 hospitalized patients in the DPC database. After removal of duplicate records, the data of 41 patients were included in the final analysis. The study population comprised 26 men (63%) and 15 women (37%) with a median age of 53 years (range, 10–89 years). The median length of hospital stay was 13 days (range: 2–157 days). The detailed baseline characteristics are summarized in Table 1. With respect to organ involvement, ocular manifestations were the most frequent presentation observed in 17 patients (41%). Pulmonary involvement was identified in 7 patients (17%), hepatic involvement in 5 (12%), and other organ involvement in 2 (5%). For 10 patients (24%), the specific organ involvement could not be clearly determined from the available records. Diabetes mellitus was the most frequently recorded comorbidity (7 patients, 17%), followed by hypertension (2 patients, 5%). Other comorbidities were infrequent. Albendazole therapy was administered to 22 patients (54%). The median duration of albendazole treatment during hospitalization was 3 days (range, 1–19 days). Diethylcarbamazine was administered to 1 patient (2%) for 3 days. Surgical management was primarily ophthalmologic. Pars plana vitrectomy was performed for 10 patients (24%), and intraocular lens implantation (lens reconstruction) was performed for 8 patients (20%). Glaucoma filtration surgery was performed for 1 patient (2%).

**Table 1:** Characteristics of hospitalized patients with toxocariasis (*n* = 41).

Characteristics	Total (*n* = 41)
Clinical outcomes	
Length of hospital stay, days, median (range)	13 (2–157)
Demographic characteristics	
Age, years, median (range)	53 (10–89)
Male sex, n (%)	26 (63%)
Height, cm, mean (SD)	160.5 (8.4)
Weight, kg, mean (SD)	57.2 (13.7)
Body mass index, kg/m^2^, mean (SD)	22.0 (4.1)
Organ involvement	
Eye	17 (41%)
Lung	7 (17%)
Liver	5 (12%)
Other	2 (5%)
Unknown	10 (24%)
Comorbidities	
Diabetes mellitus	7 (17%)
Hypertension	2 (5%)
Cognitive impairment / dementia	2 (5%)
Dyslipidemia	1 (2%)
Arrhythmia	1 (2%)
Congestive heart failure	1 (2%)
Old cerebral infarction	1 (2%)
Treatments	
Albendazole administration	22 (54%)
Albendazole treatment duration, days, median (range)	3 (1–19)
Diethylcarbamazine administration	1 (2%)
Diethylcarbamazine treatment duration, days	3
Surgical procedures	
Pars plana vitrectomy	10 (24%)
Intraocular lens implantation (lens reconstruction)	8 (20%)
Glaucoma filtration surgery	1 (2%)

Data are presented as mean (SD, standard deviation), median (range), or number (%).

### 3.2 Geographic distribution and demographic features

The regional and prefectural distributions of patients hospitalized for toxocariasis are shown in Fig. 1. The largest proportion of patients resided in the Kinki region (13 cases, 32%), followed by the Kyushu–Okinawa (12 cases, 29%) and Kanto regions (8 cases, 20%). Cases were sporadically distributed in other regions, and no patients were identified from Hokkaido or Tohoku during the study period. The mean number of hospitalized patients during the study period was 5.9 cases per year (Fig. 2A). The age distribution of patients is shown in Fig. 2B. The highest number of cases was observed in patients aged 70–79 years (8 patients, 20%), followed by those aged 50–59 years (7 patients, 17%). None of the patients were younger than 10 years.

**Fig. 1:**
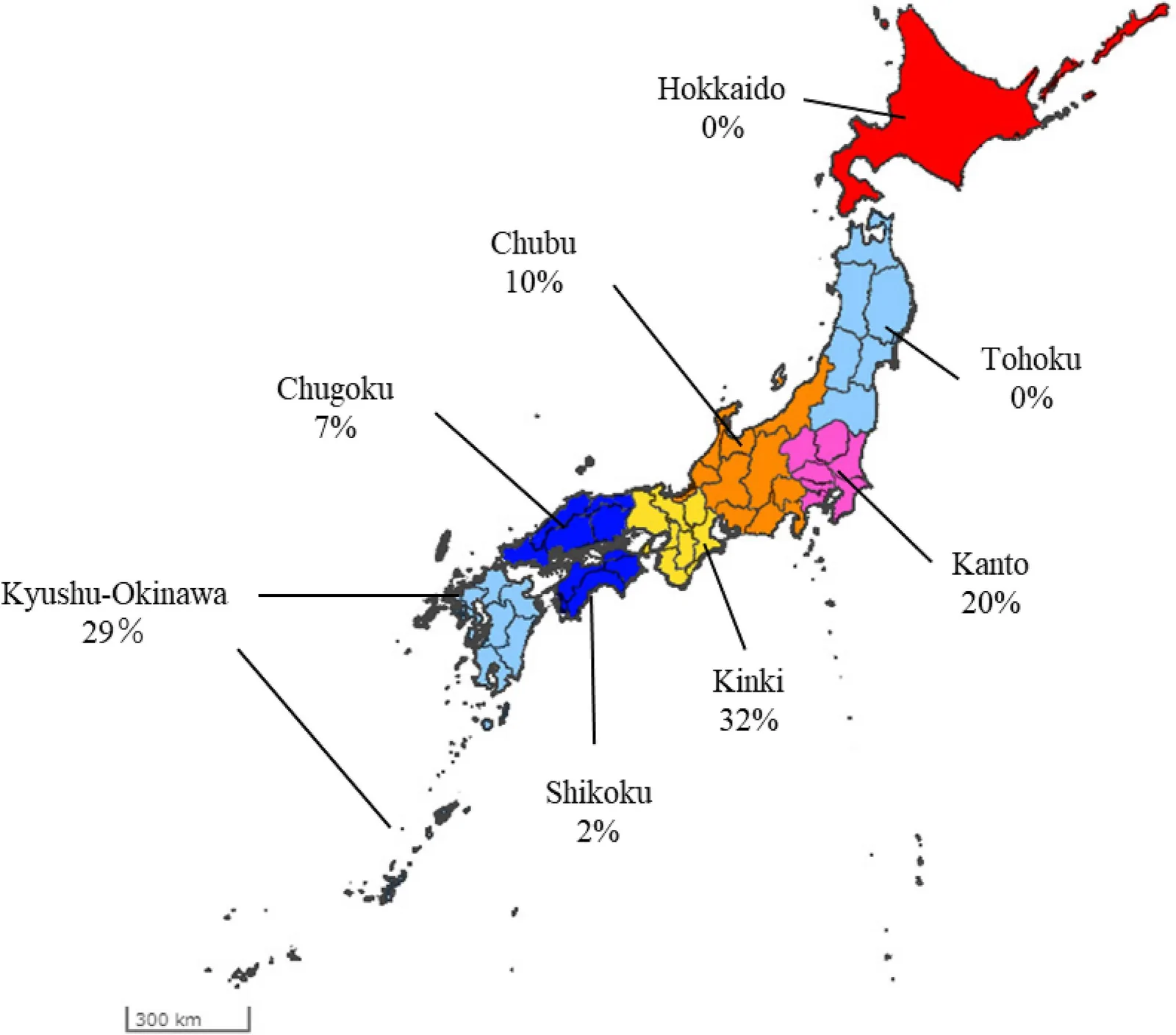
Geographic distribution of patients hospitalized for toxocariasis in Japan. The map shows the regional and prefectural distributions of patients hospitalized for toxocariasis identified in the Japanese Diagnosis Procedure Combination database across the fiscal years 2014 through 2020. Patients were categorized according to their registered residential prefectures and grouped into standard administrative regions. Relative clustering of cases was observed in Western Japan, particularly in the Kinki and Kyushu–Okinawa regions, whereas no cases were identified in Hokkaido or Tohoku during the study period.

**Fig. 2:**
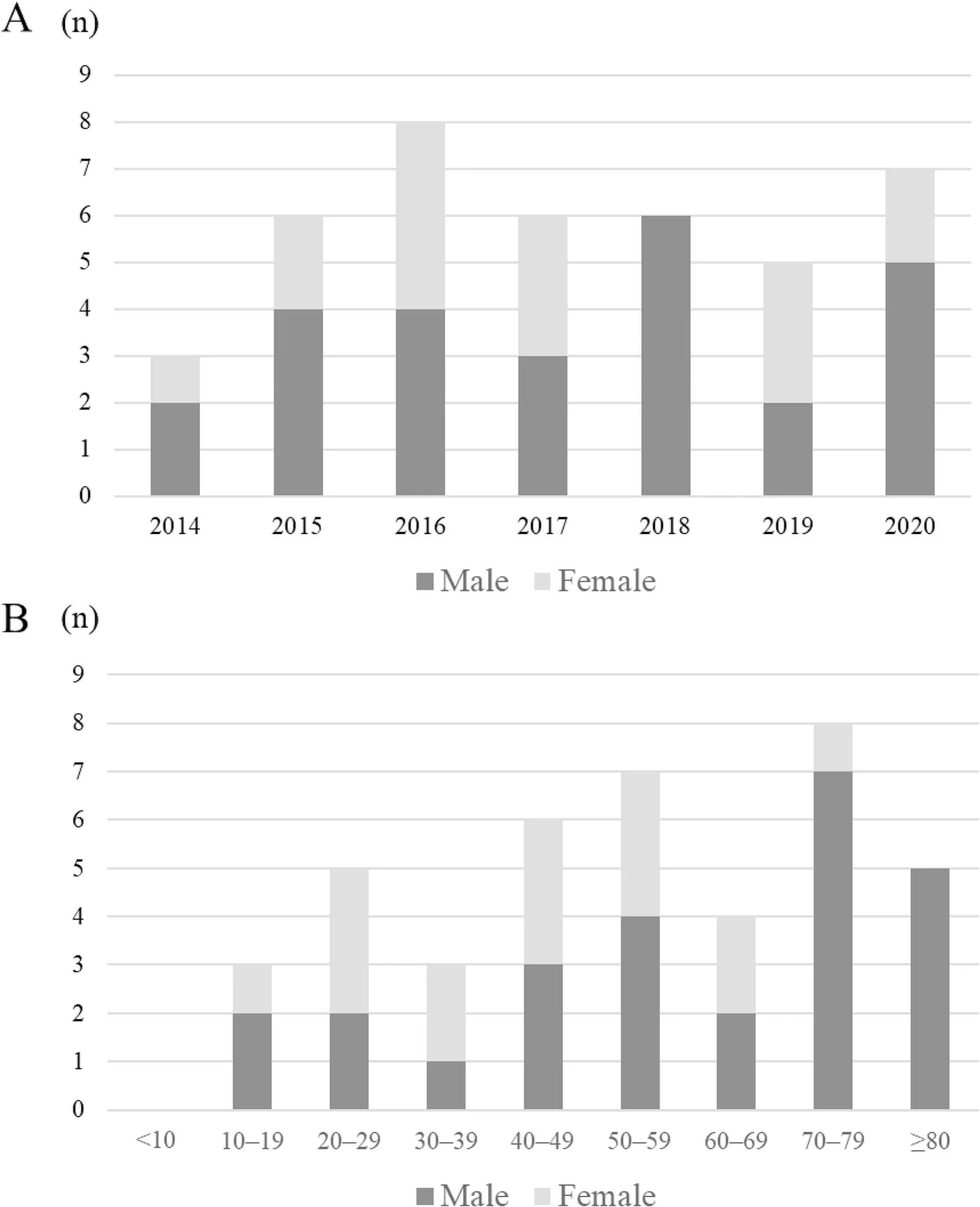
Annual and age distribution of patients hospitalized for toxocariasis in Japan. (A) Annual number of patients hospitalized for toxocariasis stratified by sex across the fiscal years 2014 through 2020. Bars represent the number of male and female patients each year. (B) Age distribution of patients hospitalized stratified by sex. Patients were categorized into 10-year age groups. The highest number of cases was observed among patients aged 70–79 years. Patients younger than 10 years were not identified in this study.

## 4 Discussion

The present nationwide DPC analysis demonstrated that hospitalized toxocariasis in Japan predominantly affects middle-aged and older adults and is frequently associated with ocular involvement requiring surgical intervention. No cases were identified in children younger than 10 years, and cases were geographically clustered in Western Japan. These findings provide the first nationwide overview of toxocariasis cases requiring hospitalization in contemporary Japan. They also likely reflect the characteristics of more severe forms of toxocariasis requiring hospitalization, particularly ocular diseases, rather than the full clinical spectrum of toxocariasis.

The absence of patients younger than 10 years and the predominance of middle-aged and older adults contrasts with the traditional view that toxocariasis is primarily a pediatric disease (Despommier, 2003; Macpherson, 2013). Previous Japanese studies on ocular toxocariasis have also reported a predominance of adult patients (Yokoi et al., 2003; Yoshida et al., 1999). In contrast, a large US survey of ocular toxocariasis reported a median age of 11.5 years (Woodhall et al., 2012), suggesting that the age distribution of patients with clinically significant ocular toxocariasis differs across geographic settings. This finding should be interpreted with caution, because pediatric VLM is often mild and managed in outpatient settings, whereas adult ocular toxocariasis more frequently requires hospitalization and surgical intervention, thereby increasing the likelihood of inclusion in an inpatient database. Additionally, pediatric hospitals are less frequently included in the DPC system, which may further contribute to the underrepresentation of hospitalized pediatric cases. Nationwide seroprevalence data for *Toxocara* infection in Japan are lacking. An earlier seroepidemiological study conducted in Yamaguchi Prefecture reported seropositivity rates of 3.1% in children and 3.7% in adult women, with regional variation among urban, rural, and fishing areas (Matsumura and Endo, 1983). However, these historical regional data may not reflect the current nationwide epidemiology of toxocariasis in Japan. Nevertheless, the predominance of adult male patients suggests that the exposure patterns in Japan may differ from the classical soil-mediated transmission paradigm. Previous Japanese studies have provided clinical and parasitological evidence supporting foodborne transmission through raw or undercooked chicken as a paratenic host (Morimatsu et al., 2006; Okada et al., 2021). The adult-dominant pattern observed in this nationwide cohort is consistent with this hypothesis.

In the present study, hospitalized cases were concentrated in the Kinki region (32%) and Kyushu–Okinawa region (29%), indicating distinct geographic clustering. This westward predominance may reflect regional differences in exposure or case detection; however, given the small number of cases, these geographic patterns should be interpreted cautiously. Although causality cannot be established from the present study, one plausible explanation is local dietary practices, including the consumption of raw or undercooked chicken meat or liver (Morimatsu et al., 2006; Okada et al., 2021), which have been implicated in foodborne transmission in Japan. At the same time, regional differences in physician awareness and diagnostic practices, particularly in Kyushu, where parasitic diseases are relatively well-recognized, may have increased case detection, introducing potential surveillance bias. Therefore, the geographic distribution observed in this study likely reflects both regional exposure patterns and variations in the diagnostic intensity. In higher-incidence regions, careful dietary history taking and targeted public health education may be important to reduce the number of severe cases requiring hospitalization.

The treatment patterns identified in this study suggest a discrepancy between the recommended therapeutic regimens and inpatient management as captured in the DPC database. In Japan, long-term administration of albendazole has been reported to be safe and effective for larva migrans syndrome (Hombu et al., 2019). However, the median duration of albendazole therapy during hospitalization in our cohort was only 3 days. This short inpatient duration likely reflects routine clinical practice. Patients with ocular toxocariasis often undergo surgical intervention and are discharged once their condition stabilizes, whereas patients with VLM are discharged after the improvement of acute symptoms with continuation of anthelmintic therapy in an outpatient setting (Wakabayashi et al., 2021). Adult toxocariasis, particularly ocular disease, can result in irreversible visual impairment (Woodhall et al., 2012), and appropriate therapeutic management is clinically important. However, the total duration of therapy and post-discharge management could not be evaluated because the DPC database only captures medications administered during hospitalization. Therefore, the inpatient treatment duration observed in this study should be interpreted with caution, as it may not reflect the full course of therapy.

The limitations inherent to the use of administrative databases should be acknowledged. First, this study included only patients who had been hospitalized and did not include mild cases managed in outpatient settings or asymptomatic infections. Consequently, the true prevalence of toxocariasis in Japan is likely to be higher than that in the present inpatient cohort. In addition, ocular toxocariasis is more likely to require hospitalization and surgical intervention, and our findings may have been disproportionately influenced by ocular diseases. Second, the DPC database does not contain detailed clinical information, such as dietary history, exposure to animals, or serological data (e.g., ELISA antibody titers). Consequently, the source of infection and diagnostic accuracy of individual cases could not be directly verified. In addition, the diagnoses were identified based on ICD-10 codes, which may be subject to misclassification. Despite these limitations, this nationwide analysis provided valuable insights into the epidemiological characteristics and clinical profiles of toxocariasis patients requiring hospitalization in Japan, highlighting the predominance of adult cases and the burden of ocular involvement. These findings provide important baseline data for understanding severe toxocariasis and may inform future epidemiological studies and preventive strategies.

## 5 Conclusion

In conclusion, this nationwide analysis using the Japanese DPC inpatient database demonstrated that toxocariasis requiring hospitalization in Japan predominantly affects middle-aged and older adults and is frequently associated with ocular involvement requiring surgical intervention. The geographic clustering observed in Western Japan and the adult-dominant demographic pattern may reflect differences in exposure characteristics from the classical pediatric soil-mediated paradigm, although these findings should be interpreted cautiously. While limited to inpatient data, this study provides the first comprehensive overview of patients hospitalized for toxocariasis in Japan reflecting more severe forms of the disease.

## Declaration of generative AI and AI-assisted technologies in the manuscript preparation process

During the preparation of this manuscript, the authors used ChatGPT (OpenAI) to assist with improving the English language and enhancing the readability of the manuscript. The authors critically reviewed and edited all AI-generated content, take full responsibility for the accuracy and integrity of the manuscript, and approve its final version.

## CRediT authorship contribution statement

**Takafumi Matsunaga:** Writing – original draft, Visualization, Data curation. **Naoaki Ohkubo:** Writing – review & editing, Data curation. **Kei Yamasaki:** Writing – review & editing, Writing – original draft, Visualization, Supervision, Methodology, Formal analysis, Data curation. **Masahiro Tahara:** Writing – original draft, Visualization, Data curation. **Kei Nakamura:** Writing – review & editing. **Hiroaki Ikegami:** Writing – review & editing. **Konomi Sennari:** Writing – review & editing. **Takanobu Jotatsu:** Writing – review & editing. **Chinatsu Nishida:** Writing – review & editing. **Makoto Okawara:** Writing – review & editing, Data curation. **Yoshihisa Fujino:** Writing – review & editing, Data curation. **Shinya Matsuda:** Writing – review & editing. **Kiyohide Fushimi:** Writing – review & editing. **Hiroshi Mukae:** Writing – review & editing. **Kazuhiro Yatera:** Writing – review & editing, Supervision.

## Ethics approval

This study was approved by the Institutional Review Board of the University of Occupational and Environmental Health, Japan (approval number: R4-045). The requirement for informed consent was waived owing to the retrospective nature of the study and the use of anonymized administrative data.

## Funding

This study did not obtain any specific grants from funding bodies in the public, commercial, or not-for-profit sectors.

## Declaration of competing interest

The authors declare that they have no known competing financial interests or personal relationships that could have appeared to influence the work reported in this paper.

## Data Availability

The data analyzed in this study were obtained from the Diagnosis Procedure Combination (DPC) database through the DPC Research Institute. The data are not publicly available because of restrictions on data sharing and cannot be provided by the authors.
